# Mechanical Properties of Colorectal Cancer Cells Determined by Dynamic Atomic Force Microscopy: A Novel Biomarker

**DOI:** 10.3390/cancers14205053

**Published:** 2022-10-15

**Authors:** M. Manuela Brás, Tânia B. Cruz, André F. Maia, Maria José Oliveira, Susana R. Sousa, Pedro L. Granja, Manfred Radmacher

**Affiliations:** 1 Instituto de Investigação e Inovação em Saúde (i3S), Universidade do Porto, 4200-135 Porto, Portugal; 2 Instituto de Engenharia Biomédica (INEB), Universidade do Porto, 4200-135 Porto, Portugal; 3 Faculdade de Engenharia da Universidade do Porto, 4200-465 Porto, Portugal; 4 Instituto de Biologia Molecular e Celular (IBMC), Universidade do Porto, 4200-135 Porto, Portugal; 5 Instituto de Ciências Biomédicas Abel Salazar (ICBAS), Universidade do Porto, 4050-313 Porto, Portugal; 6 Instituto Superior de Engenharia do Porto (ISEP), Instituto Politécnico do Porto, 4200-072 Porto, Portugal; 7 Institute of Biophysics, University of Bremen, 28334 Bremen, Germany

**Keywords:** colorectal cancer (CRC), atomic force microscopy (AFM), frequency sweep, viscoelasticity, creep, stress relaxation, power-law exponent, focal adhesions

## Abstract

**Simple Summary:**

Colorectal cancer (CRC) is presently the third-most abundant and the second-most lethal cancer worldwide. Thus, there is a real and urgent need to investigate the processes behind the appearance, development, and proliferation of CRC cells. Several biochemical pathways have been investigated to understand their role in oncogene activation and tumor-suppressor gene inhibition. Despite the research increase in biochemistry, there is still a need to better understand the biophysical cues that drive the activation of signaling pathways relevant to mechanotransduction and cell transformation. The elucidation of these biological processes may help to hinder oncogenic mechanisms and to find biomarkers that could be used to design more personalized therapeutic strategies.

**Abstract:**

Colorectal cancer (CRC) has been addressed in the framework of molecular, cellular biology, and biochemical traits. A new approach to studying CRC is focused on the relationship between biochemical pathways and biophysical cues, which may contribute to disease understanding and therapy development. Herein, we investigated the mechanical properties of CRC cells, namely, HCT116, HCT15, and SW620, using static and dynamic methodologies by atomic force microscopy (AFM). The static method quantifies Young’s modulus; the dynamic method allows the determination of elasticity, viscosity, and fluidity. AFM results were correlated with confocal laser scanning microscopy and cell migration assay data. The SW620 metastatic cells presented the highest Young’s and storage moduli, with a defined cortical actin ring with distributed F-actin filaments, scarce vinculin expression, abundant total focal adhesions (FAK), and no filopodia formation, which could explain the lessened migratory behavior. In contrast, HCT15 cells presented lower Young’s and storage moduli, high cortical tubulin, less cortical F-actin and less FAK, and more filopodia formation, probably explaining the higher migratory behavior. HCT116 cells presented Young’s and storage moduli values in between the other cell lines, high cortical F-actin expression, intermediate levels of total FAK, and abundant filopodia formation, possibly explaining the highest migratory behavior.

## 1. Introduction

According to the Global Cancer Observatory report (GLOBOCAN 2020 report) published by Sung et al., breast cancers are the most frequent tumors (11.7%), followed by lung (11.4%) and colorectal cancers (CRC; 10.0%) [1]. Interestingly, although CRC ranks as third in terms of incidence, it is second in terms of mortality [2].

The development of CRC is related to a deficiency in cell migration from the crypt, which depends on the adenomatous polyposis coli (APC) protein, leading to the accumulation of cells in the amplified zone of the colonic crypt. The amount of these cells may increase exponentially by accumulating mutations, thus resulting in the formation of a tumor [3]. In CRC, the epithelial-to-mesenchymal (EMT) transition drives cellular migration and is characterized by the acquisition of a mesenchymal phenotype through tight junction dissolution, disruption of apical-basal polarity, and reorganization of the cytoskeletal architecture [4]. The neoplastic process relies not only on the referred molecular biology and biochemistry phenomena but also on internal and external forces exerted by the cells that are translated into biochemical signals (mechanotransduction) [3]. A deeper understanding of the biophysical mechanisms behind biochemical pathways can help to clarify cell motility, invasion, migration, extravasation, and metastization processes and thus assist in the development of new and improved therapeutic strategies [5].

To perform mechanobiology and mechanotransduction studies, several techniques can be used, namely, atomic force microscopy (AFM), microindentation, shear rheometry, shear wave, magnetic resonance elastography, micropipette aspiration, optical stretching and/or magnetic twisting cytometry, all of which have been developed to evaluate the mechanical properties of cells and tissues [6]. AFM can be carried out in physiological conditions and hence allows not only the determination of mechanical characterization of samples, including cells [6,7] but also measurement directly on tissues [8]. For instance, Boccaccio et al. used nanoindentation by AFM to study the effect of surface roughness and cell shape in mechanical properties variation of SW480 and SW620 cell lines on colon cancer and lymph-node-derived metastases [9].

Previous research reported the quantification of cells’ mechanical properties considering that they are isotropic, purely elastic, and semi-infinite materials, presenting the determination of the apparent Young’s modulus since the viscous component cannot be separated from the elastic one [10]. However, cells are structures that also show a viscous response. The cytoskeletal filaments and the cytosol determine the elastic and viscous properties of cells. The polymerization of monomeric G-actin to filamentous F-actin affects the elastic properties of cells. Viscosity seems to depend on the complexity of the cytosol due to the concentration of several components and the presence of a polymeric network and organelles. The power-law exponent of the storage modulus is often interpreted as an expression of cell fluidity and the solid-like or liquid-like state of cells [11]. Hence, we used AFM frequency sweep to quantify the viscoelasticity of cells based on the determination of the frequency-dependent storage and loss moduli. A power-law exponent was applied to the storage modulus, which, if often correlated with the fluidity of the cell cytosol, as well as a liquid-like state, facilitates the shape changes and cell deformation, thereby promoting the invasion of the basal lamina, underlying stroma and vascular and lymphatic spaces [12]. Furthermore, this methodology can also provide information about creep and stress relaxation as a function of time [11,13]. Stress–relaxation is a time-dependent component, decreasing under a constant strain, and creep is defined as constant stress with decreasing strain as a function of time [14]. Herein, we aimed to unveil the mechanical properties of different CRC cell lines by different atomic force methods and cross-analyzing such results with cell-migration features, namely, cytoskeleton protein expression and localization and gap-closure abilities. Hence, we quantified the mechanical properties using dynamic AFM on the three CRC cell lines: HCT15, HCT116, and SW620. Frequency sweep results showed that SW620 was the stiffest, also presenting a lessened migratory behavior. HCT15 cells were the softest, exhibiting abundant focal adhesions and presenting a rapid migratory behavior. HCT116 cells, which in terms of stiffness exhibited an intermediate level, presented filopodia formation and the highest migratory behavior. In summary, our study elucidates the mechanical properties of CRC cells and can potentially be used as a biomarker for their motility and migratory behaviors.

## 2. Materials and Methods

### 2.1. CRC Cell Cultures

The culture of the CRC cell lines HCT116 and HCT15 (derived from a primary colorectal cancer) and SW620 (derived from lymph-nodes metastasis) was carried out in T25 flasks. HCT116 and HCT15 cells were cultured in Roswell Park memorial Institute medium (RPMI) 1640 (Pan-BioTech, Aidenbach, Germany) supplemented with 10% (*v*/*v*) fetal bovine serum (FBS; Gibco, Grand Island, NE, USA) and 1% (*v*/*v*) penicillin/streptomycin (pen/strep; Merck, Darmstadt, Germany).

The SW620 cell line was cultured in Dulbecco’s modified Eagle medium (DMEM; Pan-Biotech, Aidenbach, Germany) supplemented with 10% (*v*/*v*) FBS and 1% (*v*/*v*) pen/strep. Before medium supplementation, FBS was inactivated at 56 °C for 30 min and filtered with a 0.22 µm filter system. After cell confluence, the monolayer was washed with 5 mL of phosphate-buffered saline (PBS). The PBS was removed, and 1 mL of a mixture of 0.05% (*v*/*v*) trypsin/0.53 mM ethylenediamine tetra acetic acid (EDTA; Merck, Darmstadt, Germany) was added. The cells were incubated in 5% CO_2_ for 3 min. After confirming the microscope detachment of all cells, 5 mL of each medium (RPMI1640 or DMEM, depending on cell type) was added. Cells were then centrifuged at 1300 rpm for 5 min. Then, 5 × 10^4^ and 7.5 × 10^4^ cells were seeded in round Petri dishes 55 mm in diameter (TPP, Darmstadt, Germany). The two densities were used to determine the best condition for mechanical quantifications using the AFM. Cells were kept inside the CO_2_ incubator for 24 or 48 h before AFM analysis. In the case of SW620 cells, the measurements were performed after 4 days since shorter periods did not promote adequate cell attachment to the Petri dish. The CRC cell lines were observed in cell clusters.

### 2.2. Assessment of Cell Mechanics Using Static and Dynamic AFM Methodologies

An MFP-3D atomic force microscope (Asylum Research, Santa Barbara, CA, USA), mounted on an inverted optical microscope (Axiovert 135TV, Zeiss, Aalen, Germany), was used to quantify the mechanical properties of CRC cells. The optical microscope combined with the atomic force microscope provided adequate observation of the tips and cells. The Petri dish with cells was fixed to an aluminum holder with vacuum grease and mounted on the atomic force stage with 2 magnets. The atomic force microscope head, including the sample, was enclosed in custom-built polymethylmethacrylate (PMMA) box to inject and maintain a 5% CO_2_ environment. Force–distance curves were recorded above the nuclear region of the cells, typically at a scan rate of 2 Hz, corresponding to a maximum loading rate of 1 nN/s. The trigger threshold was set to 3 nm (maximum deflection of the cantilever); however, due to tilt in the force curve (in the part off the surface), the maximum deflection was often up to 7 nm, which corresponds to a maximum loading force of 1 nN. The mechanical properties (Young’s modulus) were obtained by fitting the data between 0.5 nm and 3 nm in deflection (corresponding to 0.1 nN to 0.4 nN).

Conventional force–distance curves were recorded in at least 70 cell clusters for each CRC cell. Force mappings of 6 × 6 force–distance curves were acquired over a cell area of 5 × 5 μm^2^.

The measurement of single force–distance curve maps and frequency sweep maps were obtained following this order for all cell lines. The atomic force microscope measurements were always performed after the cells’ second passage.

All data analysis and processing were performed using the package IGOR Pro 7 (Wavemetrics, Tigard, OR, USA) to quantify the mechanical properties of cells. Hertz’s model for spherical tips was used to calculate Young’s modulus for each force–distance curve within a force map [15]. Hertz’s model equation considers the geometry parameters of the cantilever tip (regarding the shape and cantilever tip radius). The median of these values (rather than the mean, due to the asymmetry of the distribution of the values) was considered as a representative modulus of a force volume, thereby of each cell cluster region. The parameters used in Hertz’s fit window were the following: spring constant of the pre-calibrated cantilever provided by the supplier (k); the fit model was a sphere; the tip radius used was 75 nm; fit range was set from 500 pm to 2 or 3 nm deflection; tilt correction of the force–distances curves were performed; Poisson’s ratio used was 0.5. The deflection sensitivity was obtained by fitting a thermal to the thermal data.

#### 2.2.1. Calibration Procedure

Pre-calibrated peak force cantilevers (model PFQNM-LC-A-CAL; Bruker, Billerica, MA, USA) with a pre-defined nominal spring constant were used. A thermal of the cantilever was captured in deionized water to obtain the power spectral density (PSD) of the deflection data. The thermal was fitted with the single harmonic oscillator (SHO) or the Lorentzian model. The selected model was the Lorentzian one since it better fit the thermal acquired in liquids. For instrumental reasons, the data were captured with a deflection sensitivity based on an SHO fit, which was then corrected before analysis based on a Lorentzian fit (see Supplementary Materials, Figure S1) [16]. The Petri dishes with deionized water were fixed to an aluminum holder with vacuum grease and mounted on the atomic force microscope stage with two magnets. The inverse optical lever sensitivity (InvOLVS) of the cantilever was calculated using the no-touching calibration method. For frequency sweep, the hydrodynamic correction factor (b-hydrodynamics factor) of the cantilever was measured in each culture medium used following the procedure by Alcaraz et al. [17].

#### 2.2.2. Single Force–Distance Curve Maps

**Figure 1:**
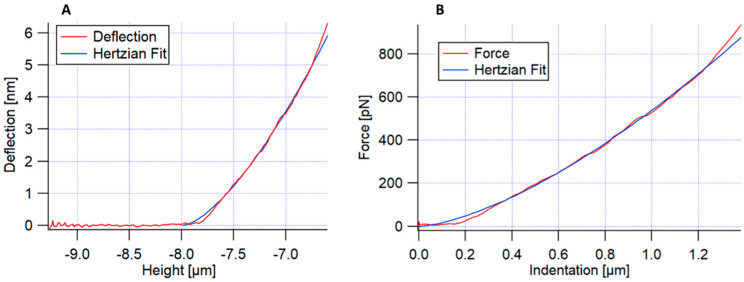
Representation of an example of the approach of a conventional force–distance curve. (**A**) Approach curve (cantilever deflection versus the height piezo), with Hertz’s fitting; (**B**) Hertz’s fitting of the force versus indentation of the sample.

The force–distance curves were performed on HCT15, HCT116, and SW620 cells. The measurements were carried out while always keeping constant the maximum applied force, meaning that the value of indentation was variable since cells showed variable elastic modulus values. Conventional force–distance curves were recorded, measured in at least 70 cell clusters, meaning that the value of cell cluster indentation was variable. As before, force mappings of 6 × 6 force–distance curves were acquired over an area of 5 × 5 μm^2^. In this method, the apparent Young’s modulus was calculated by applying Hertz’s model fitting to the approach curve [18]. Figure 1 presents an example of a conventional force–distance curve obtained for each pixel of each force map. Figure 1A shows the approach curve (cantilever deflection versus the height piezo), and the deflection approach curve fitting, using Hertz’s model. Figure 1B shows Hertz’s fitting of the force versus the indentation of the sample.

#### 2.2.3. Frequency Sweep

**Figure 2:**
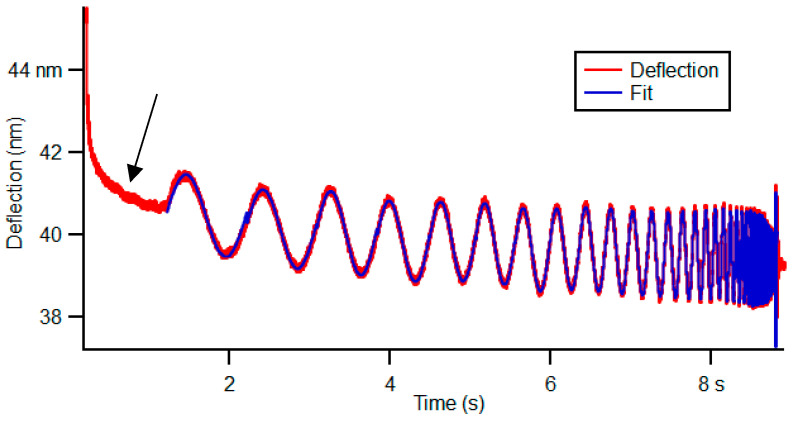
A representative of the deflection signal versus time during dwell, i.e., while the tip is in contact for 8.7 s, during frequency sweep. The first region of red curve (identified by the black arrow) presents the sample creep after loading the cell during the approach. This stress relaxation of the cell was not analyzed. The analysis of the curve starts with the curve fitting (blue color).

Quantification of dynamic viscoelastic properties was performed in CRC cells using frequency sweep. During cell indentation by the cantilever, a sinusoidal excitation signal is applied to the z-piezo transducers. The cantilever experienced in-phase and out-of-phase deflection signals, which are used to determine the complex shear modulus, creep compliance, and stress relaxation, as described well by de Sousa et al. [11]. In the present work, at the end of a conventional force–distance curve, a frequency sweep between 1 and 100 Hz, for 8.7 s, was applied with a trigger point of 3 nm and an amplitude of 80 nm [11,13]. At least 70 cell clusters were measured, with a dwell time of 8.7 s. Maps of 5 × 5 μm^2^ were measured, with 16 force–distance curves using the frequency sweep method, which allowed the measurement of the power-law exponent (storage modulus as a function of frequency). It was also possible to observe the creep and stress relaxation. An example of a frequency sweep curve is presented in Figure 2, presenting the initial creep and stress relaxation of the sample, although the initial creep and stress relaxation were not analyzed. Basically, using frequency sweep, the cantilever oscillated around a given indentation with a certain amplitude at a given frequency. When the cantilever contacts the cell, the force increases quickly until the trigger point (maximum cantilever deflection defined) is reached. The cantilever is excited to sinusoidal oscillations with frequencies from 1 to 100 Hz for the 8.7 s in contact. We used 50 frequencies, which were spaced in a geometric series (1 Hz, 1.15 Hz, 1.32 Hz, …. 1000 Hz), such that we had a constant density of values on a logarithmic frequency scale. If the cantilever touches a hard or elastic sample and its excitation is harmonic but off-resonance on the basis, then the deflection signal and the sinusoidal excitation signal are in-phase. If the cantilever touches a viscous sample with the excitation in the same way, then it exhibits its maximum deflection when moving fast through the fluid. In the present case, there is the inflection point of the sinusoidal excitation, meaning that in the viscous part of the sample, the deflection and excitation are 90° out-of-phase. In summary, if the sample behaves more solid-like or more liquid-like, it can be expressed by the phase shift between the two signals, with values between 0° and 180°, respectively [19].

Cells can present creep and stress relaxation as a function of time [11]. Figure 2 shows an example of the frequency sweep curve, where it can be observed that when the tip indents the sample in the first seconds, the sample presents creep and stress relaxation as a function of time. However, although viscoelastic materials such as cells present creep and stress relaxation, these parameters were not analyzed in this work [20].

To analyze the data, several parameters were used: spring constant k of the pre-calibrated cantilever provided by the supplier, Poisson’s ratio of 0.5, sphere tip radius of 75 nm, analysis range from 500 pm to 3 nm deflection, the correction factor obtained in the PSD corresponding to the fit thermal with the Lorentz’s model, and finally, the b-hydrodynamic factor of 7.43 × 10^−7^.

**Figure 3:**
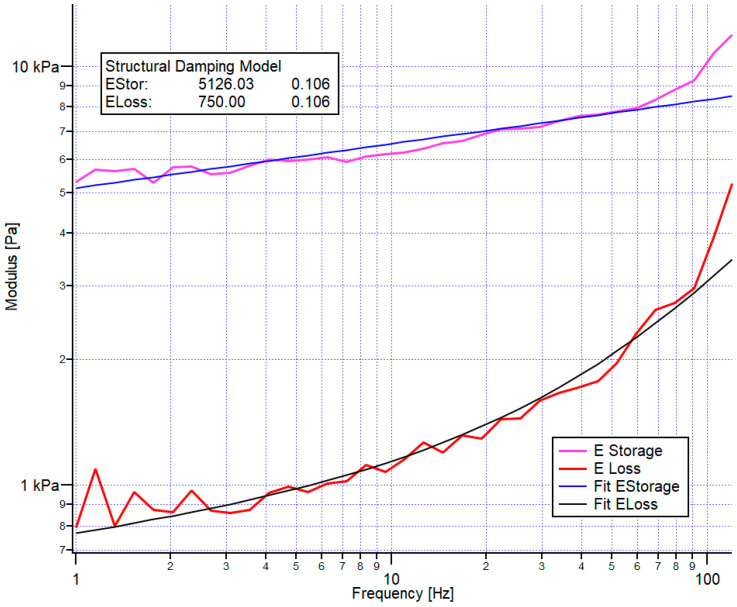
An example of the loss and storage modulus versus frequency obtained by the frequency sweep. A power-law fit is applied to the storage modulus, whereas the structural damping model is used to fit the loss modulus. Both fit procedures result in a storage and loss modulus value for the lowest frequency (here, 1 Hz). Presently, 5126 Pa and 750 Pa are shown, respectively, and a power-law exponent of 0.106.

For each frequency, we obtained the loss and the storage moduli, as presented in Figure 3. Since the storage modulus follows a power law, we characterized the storage modulus by its value at the lowest frequency (here, 1 Hz) and the power law in the example of Figure 3. The storage modulus and its power law were 5126 Pa and 0.106, respectively. Despite applying a correction for the hydrodynamic drag of the cantilever, the apparent loss modulus showed, at a higher frequency, additional viscous damping, which was attributed to the viscosity of the medium. Thus, we applied a double power-law fit to the data (lower exponent determined from the elastic modulus, plus a second one corresponding to hydrodynamic damping- Exponent 1).

#### 2.2.4. b-Hydrodynamic Cantilever Factor Determination

Hydrodynamic correction has been performed according to Alcaraz et al. [17]. In short, we have recorded the response of the cantilever at various distances from the sample (0, 200, 300, 600, 1100, 2100, 3600, and 5100 nm), while the z-height of the sample is modulated at several amplitudes (50, 100, 200, and 500 nm) in DMEM or RPMI 1640 culture media; the viscosity of these media may be different, as well as by the viscous component of the cantilever. Following the procedure of Alcaraz et al., we extrapolated the b-hydrodynamic correction factor to a distance of 0 nm from the sample and used this correction when fitting frequency sweep data on cells [17].

### 2.3. Immunohistochemistry

Cells were seeded onto UV-sterilized glass coverslips at a concentration of 2 × 10^5^ cells per well of a 24-well plate. After 48 h of incubation, the medium was removed, and cells were washed once with PBS and fixed with 4% (*v/v*) paraformaldehyde (PFA; Electronic Sciences Microscopy, Hatfield, PA, USA) for 20 min at room temperature (RT). Cells were then washed 3 times with PBS for 5 min and permeabilized with 0.25% (*v/v*) triton X-100 (ITW, Camdenton, MO, USA) for 5 min at RT. Samples were washed 3 times with PBS and incubated with 5% (*w*/*v*) bovine serum albumin (BSA; VWR International, Radnor, PA, USA) for 1 h at RT. Subsequently, samples were incubated with the primary antibodies against total FAK polyclonal antibody (1:100; Thermofisher Scientific, Waltham, MA, USA), vinculin Ab(42H89L44) ABfinity™ rabbit MAb (1:200; Thermofisher Scientific, Waltham, MA, USA), and tubulin (1:4000, Sigma-Aldrich, Albuch, Germany) at 4 °C overnight. Secondary antibody goat anti-rabbit alexa fluor 488 F(ab′)_2_ fragment (1:100; Thermofisher Scientific, Waltham, MA, USA) was added and incubated for 1 h at RT. After 3 washing steps, the conjugated probe phalloidin alexa fluor 568 (1:40 dilution; Molecular Probes-Invitrogen, Eugene, OR, USA) was added for 30 min at RT. The nuclei were counterstained with 4′,6-diamidino-2-phenylindole dihydrochloride (DAPI; 1:500; Sigma-Aldrich, St. Louis, MI, USA), and coverslips were then mounted in vectashield (Vector Laboratories, Newark, CA, USA). Samples were analyzed by confocal laser scanning microscopy (CLSM; model SP5 microscope; Leica, Wetzlar, Germany). The imaging data were treated with ImageJ/Fiji (version 1.53C) [21].

### 2.4. Cell Migration Assays

Cell migration assays of the three CRC cells were performed in 3 independent experiments, each with 3 technical replicates per cell type. Culture inserts to wells for self-insertion (Ibidi, Graefelfing, Germany) were plated in 12-well plates (Falcon, Nürburgring, Germany). The cell culture in the inserts was used according to the manufacturer’s protocol. Each insert has two parts with outer dimensions of 8.4 mm × 8.4 mm × 5 mm. The growth area per well was 0.22 cm^2^. The coating area per well was 0.82 cm^2^, the volume per well was 70 µL, and the width of the cell-free gap was 500 µm ± 100 µm. For the migration assay, 7.5 × 10^4^ cells were used in each half of the insert. The migration was performed with an imaging system equipped with a fully automated microscope (model IN Cell Analyzer 2000; GE Healthcare, Chicago, IL, USA), with 5% CO_2_ and temperature-controlled at 37 °C for 144 h. Fifteen fields of view (FOV) with 15% overlap were acquired with a Nikon 10×/0.45 Plan Apo objective spanning the entire gap, each 4 h during the first 72 h and then each 24 h until the end of the experiment. The final images were then reconstructed with the IN Cell Developer Toolbox software (GE Healthcare, Chicago, IL, USA). Quantification of the gap closure area over time was performed with an ImageJ/Fiji macro (Wound-Healing Area v1.0) available at the “Search Engine for BioImage Analysis” repository (https://se4bia.i3s.up.pt, accessed on 25 August 2022). Averages of 2 regions of interest (ROI) per technical replicate per cell line were calculated, and the average of the 3 independent experiments has been calculated and plotted with Prism GraphPad.

### 2.5. Statistics

For the AFM analysis, the median values of Young’s modulus were determined from 3 independent experiments for each cell type. For comparing experiments, the median and the 25% and 75% percentiles to visualize differences were used; for quantifying significance of differences, the Wilcoxon Rank, which does not assume normally distributed data but rather looks at the order of data points, was used. ^(^***^)^ Denotes that the difference between groups of distinct CRC cells was statistically significant at a *p*-value below 0.001.

## 3. Results

### 3.1. Cells’ Mechanical Properties Determination by AFM

**Figure 4:**
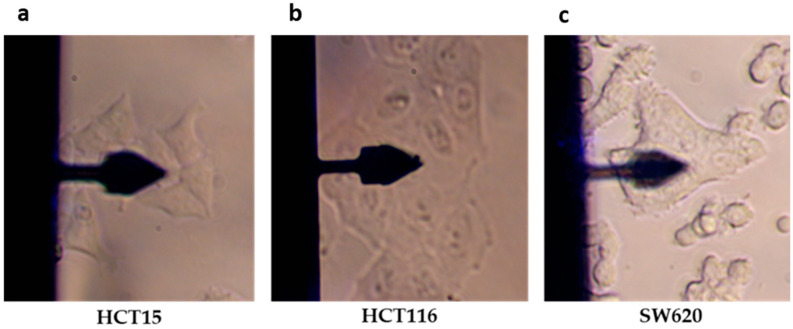
Representative images of cell types used for quantification of their biomechanical properties by AFM, using a model PFQNM-LC-A-CAL cantilever. (**a**) HCT15, (**b**) HCT16, and (**c**) SW620 cells.

To assess the mechanical properties of the CRC cell lines, cell clusters of adherent HCT15, HCT116, and SW620 cells were chosen (Figure 4). The quantification of mechanical properties was performed using conventional force–distance curve acquisition and frequency sweep.

Mechanical properties of cells were measured by AFM through the acquisition of conventional force–distance curves and force–distance curves with frequency sweep as described above. From the approach part of the force curve, we calculated Young’s modulus, which we shall call here apparent Young’s modulus since it reflects the elastic properties of the cell mainly with an additional contribution of the viscosity of the cell due to the motion of the cantilever. Frequency sweep allows separating the elastic and viscous response unambiguously, thus resulting in the storage and loss modulus as a function of frequency. The apparent Young’s modulus from the force curve ramped at typically 1 force curve per second is similar to the storage modulus of a sweep at the lowest frequency applied (1 Hz) and theoretically slightly larger.

**Figure 5:**
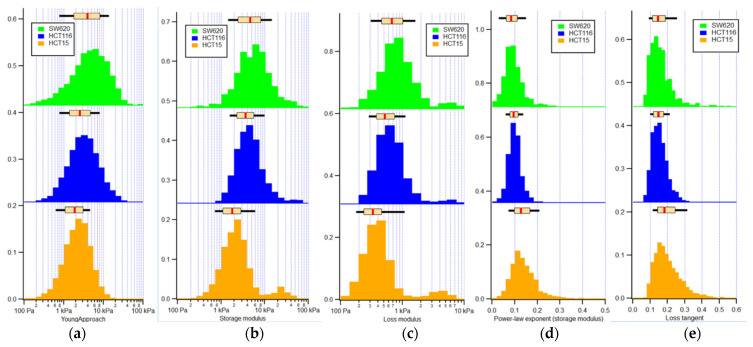
Histograms of mechanical properties quantification of the three CRC cell lines: (**a**) apparent Young’s modulus; (**b**) storage modulus; (**c**) loss modulus; (**d**) power-law exponent of the storage modulus; and (**e**) loss tangent. The boxplot above the histograms presents the median (red line) and the 25th and 75th percentiles (extent of rectangle in cream color), as well as the 10th and 90th percentiles (black lines extending beyond the box), respectively. Orange: HCT15, blue: HCT116, and green: SW620 cells.

Figure 5 shows the histograms of mechanical quantification of the three cell types, with a boxplot displaying the median and the respective percentiles (25th and 75th).

Table 1 and Table 2 present the median with the 25th and 75th percentiles along with the *p* values for the apparent Young’s, storage, loss moduli, power-law exponent applied to the storage modulus, and loss tangent of HCT15, HCT116, and SW620 cell lines.

When analyzing Table 2, significant differences can be seen regarding HCT15 and HCT116 and HCT115 and SW620 in all mechanical parameters. Comparing HCT116 and SW620 cell lines, the storage modulus, power-law exponent of the storage modulus, and the loss tangent did not present significant differences.

### 3.2. Cancer Cells’ Morphology and Motility Structures

**Figure 6:**
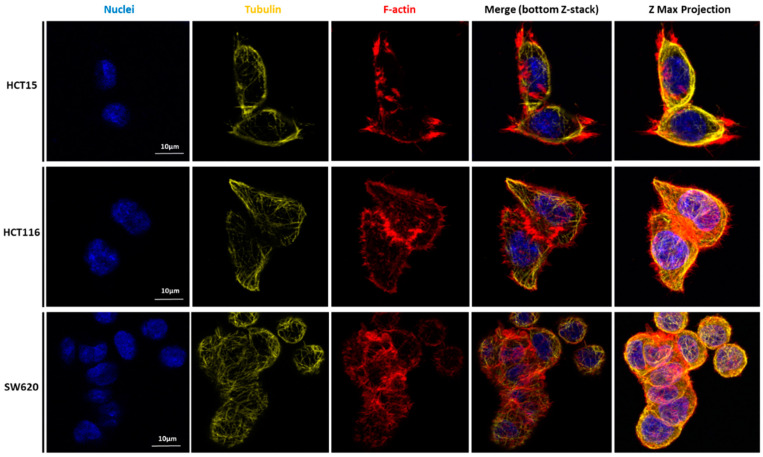
Expression of tubulin and F-actin in CRC cells. Staining of HCT15 (**top**), HCT116 (**middle**), and SW620 (**lower images**) cells for DAPI to localize the nuclei (blue), tubulin (yellow), and phalloidin to detect F-actin filaments (red). The bottom Z-stack is shown, and a Z-maximum projection of each condition is also depicted (**right panels**). Scale bars: 10 µm.

**Figure 7:**
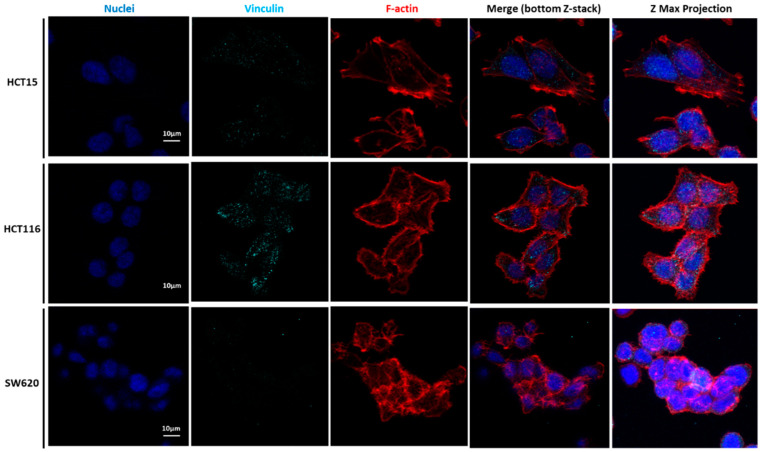
Vinculin expression in CRC cell lines HCT15 (**top**), HCT116 (**middle**), and SW620 (**lower images**) immunostained for DAPI to illustrate the nuclei (blue), vinculin (cyan), and phalloidin for F-actin filaments (red). The bottom Z-stack micrograph is shown, and a Z-maximum projection showing the overall stains is also represented (**right panels**). Scale bars: 10 µm.

**Figure 8:**
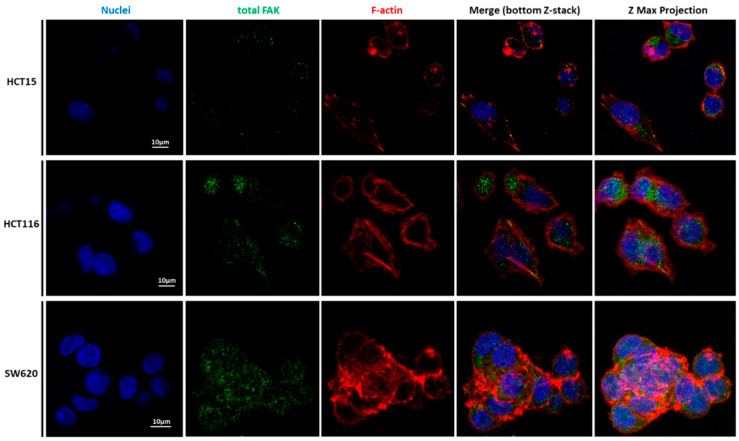
Immunolocalization of FAK in CRC cells. Staining of HCT15 (**top**), HCT116 (**middle**), and SW620 (**lower images**) cells with DAPI to identify the nuclei (blue), phosphorylated FAK (green), and phalloidin for F-actin filaments (red). Shown are the bottom Z-stack and the Z-maximum projection of the imaged area. Scale bars: 10 µm.

**Figure 9:**
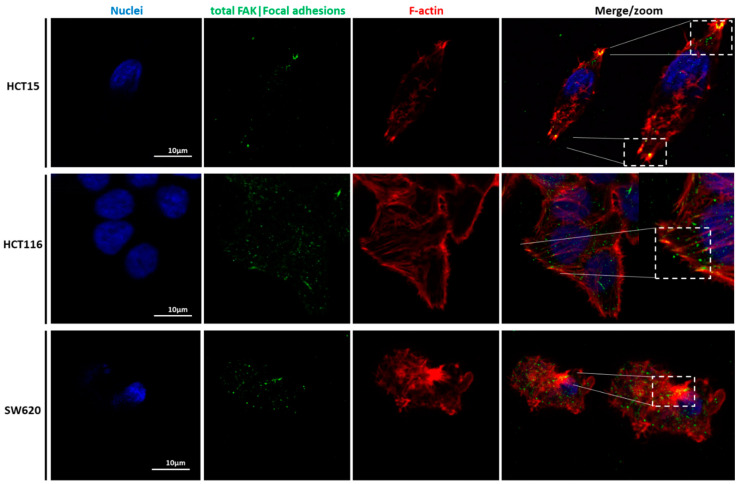
Illustration of the focal adhesion sites of CRC cells seeded in 2D. Immunolocalization of nuclei (blue), phosphorylated FAK (green), and F-actin filaments (red) in HCT15 (**top**), HCT116 (**middle**), and SW620 (**lower images**) cell lines. The zoomed areas indicate the co-localization of FAK with the F-actin filaments at the focal adhesion complexes located at the bottom z-stack. Scale bars: 10 µm.

To unveil the potential relations of CRC cell lines’ migratory features with the mechanical results, the expression and localization of cytoskeleton proteins were analyzed. Figure 6, Figure 7, Figure 8 and Figure 9 represent data obtained by CLSM, in which staining of nuclei, tubulin, F-actin filaments, vinculin, and the total FAK was performed.

Our detailed motility analysis (Figure 6, Figure 7, Figure 8 and Figure 9) revealed that HCT15 cells exhibited an elongated shape, with the absence of cortical F-actin ring but well-defined cortical tubulin cytoskeletal organization, accompanied by reduced vinculin and total FAK staining at the focal contact points, but abundant small filopodia projections, characteristic of greater migratory behavior. On the contrary, HCT116 cells exhibited an elongated shape, less-evident cortical tubulin with a well-defined cortical F-actin ring, abundant cell-cell contact areas, exhibiting vinculin and evident FAK staining, in comparison with the other cell lines, distributed throughout the whole cytoplasm. Cell margins presented abundant small filopodia, suggesting an intermediate migratory behavior.

Interestingly, SW620 cells showed a rounded shape, with a well-defined cortical F-actin, particularly evident at cell-cell contact points. These cells did not express vinculin but displayed abundant FAK, distributed throughout the cytoplasm with evident focal contact points, accompanied by the absence of filopodia, suggestive of lessened migratory behavior, as has been already suggested by Li et al. [22].

### 3.3. Cell Migration Assays

To evaluate the functional impact of the observed biomechanical properties, cell-migration assays were performed, and the graphic illustrating the gap closure as a function of time (from 0 to 144 h) was obtained for each cell line (see Supplementary Materials, Figure S2). Interestingly, our results demonstrated that SW620, the stiffest cell line, although displaying abundant FAK distributed throughout the cytoplasm, presented no filopodia motility structures and was indeed the less-migratory cell line. In contrast, HCT15 cells, which had the softest biomechanical properties, exhibited abundant focal adhesions, abundant small filopodia projections, and experienced faster migratory behavior. Importantly, HCT116 cells, which in terms of stiffness exhibited an intermediate state, presented abundant filopodia at the cell margins and displayed the highest migratory behavior

Table 3 presents a summary of the results obtained in AFM, CLSM, and cellmigration assays for the CRC cell lines analyzed, namely, HCT15, HCT116, and SW620.

## 4. Discussion

The main objective of this study was to compare the mechanical properties of three CRC cell lines, correlating their biomechanical properties with cell structure and migratory behavior.

The selection of the cells was based according to the consensus molecular subtype (CMS) classification, which included several hallmarks, such as the presence of microsatellite instability of chromosomes, mutation pathways, the inflammatory profile of the tumors, and their prognosis [2].

Accordingly, the selected CRC cell lines had one mutation in common—the Kirsten rat sarcoma (K-RAS). According to CMS classification, the HCT15 and HCT116 cells belong to the CMS 1 subtype (immune, due to the infiltration of cells from the immune system), are highly microsatellite unstable (MSI), and cancers with those cells generally present a poor prognosis [23]. The SW620 belongs to the CMS 2 subtype (canonical, ErbB, hippo-Wnt), microsatellite stable (MSS), and cancers with those cells have a good prognosis [23]. The HCT15 and HCT116 cell lines were derived from a colorectal primary tumor. They were both positive for G13D, wild-type for B-RAF, and harboring PI3Kinase (PI3K) mutations (H1047R for HCT116 and E545K; D549N for HCT15). However, while HCT116 is wild-type for TP53, HCT15 exhibits a mutation in this gene. The SW620 (metastasis at lymph node from the SW480 primary tumor cell line) cells are positive for G12V, wild-type for B-RAF and PI3K mutation, and harbor a TP53 mutation (R273H; P309S) [24].

We observed during cell culture seeding that the three cell types grew in clusters, in agreement with others, namely, for HCT15 [25], HCT116 [26], and SW620 cells [27].

We started to quantify the mechanical properties through conventional force–distance curves by calculating the apparent Young´s modulus, followed by quantification of storage modulus (elasticity) and loss modulus using frequency sweep. The apparent Young’s modulus does not allow the distinction between elasticity and viscosity since the fitting of the approach curves are fitted with Hertz’s model, which just takes into consideration the geometry and shape of the probe and the indentation, assuming that cells are isotropic materials, semi-infinite elements, and have pure elastic features [10]. One experimental way to disentangle the elastic and viscous response of cells is applying a sinusoidal modulation to the z sample height while in contact. Here, we swept the frequency of this modulation to acquire the storage modulus (G′) and the loss modulus (G″) as a function of frequency. From frequency-dependent data, we could acquire the power-law exponent in a certain frequency range (here, from 1 to 100 Hz), which several authors correlated with the cytosol cell fluidity [13] and is known to determine the ability of cells to change their shape and contract during motile processes [28,29].

The quantification of the viscoelastic properties from frequency sweep data corresponds to the slow response behavior of the cell. In this work, only one power-law exponent was measured, obtained from the frequency sweep.

The mechanical properties of the cells were also correlated with cell shape, cytoskeleton structures, the presence of focal adhesions, as well as with their migratory capability.

Through analysis of Table 3, the mechanical properties of cells present the following order for the three cell types:

- Apparent Young’s modulus: SW620 > HCT116 > HCT115;
- Storage modulus: SW620 > HCT116 > HCT115;
- Loss modulus: SW620 > HCT116 > HCT115;
- Power-law exponent of the storage modulus: SW620 < HCT116 = HCT15;
- Loss tangent: SW620 < HCT116 < HCT15.

The CRC cell lines followed the same trend in terms of apparent Young’s, storage, and loss moduli, as well as the power-law exponent of the storage modulus and the loss tangent. The apparent Young’s modulus has the same order of magnitude as the storage modulus, which can be explained due to the fact that the contribution of the storage modulus is much larger than the loss, hence the contribution of viscous effects to the response of the cell during loading in a force curve is dominated by the elastic properties and only marginally affected by the viscous properties of the cell. Both storage and loss moduli will—in a simplified picture—be related to the amount and resistance that the cytoskeleton F-actin filaments offer to deformation. The more actin filaments (including crosslinks between or bundling of actin filaments), the higher the elastic modulus will be and the larger the friction of the cytosol. When the cell is deformed by the AFM tip, the cytosol will have to be moved through the network of actin filaments. Hence, storage modulus and loss modulus will follow the same trends. Nevertheless, there are subtle differences between different cell types, which will become apparent when comparing the loss tangent (the ratio between loss and storage modulus) and power law exponent of the storage modulus, which both point to how liquid-like or how fluidic cells are. These results are corroborated by the presence of filopodia in the HCT15 and HCT116 cells, which closed the gap before the SW620 cell lines.

The studies available in the literature regarding CRC cell lines quantified the apparent Young’s modulus only. Palmieri et al. reported a value for the apparent Young’s modulus of SW620 cells of 0.480 KPa, which is below about 10 orders of magnitude of our value [30]. It can be hypothesized that the difference observed may be due to our quantification being carried out in SW620 cell clusters, where layers of F-actin filaments were observed on top of each other. Liu et al. also quantified the apparent Young’s modulus for HCT116 cells, which was in the range of 6.3 to 7.1 kPa, which is approximately three orders of magnitude higher than those reported here [26]. Nematbakhsh et al. quantified the viscoelasticity of adherent malignant breast cancer cells, namely, non-invasive MCF-7 cancer cell lines and the highly invasive MDA-MB-231 cancer cell line. The authors found an apparent Young’s modulus for MCF-7 and MDA-MB-231 cancer cell lines that is in an interval of 0.30 to 0.70 kPa and 0.10 to 0.65 kPa, respectively. Comparing the MDA-MB-231 cancer cell line, which is highly invasive, with our SW620 (the metastatic version of the SW480 primary tumor cell line), our apparent Young’s modulus is significantly higher compared with the breast cancer cell lines. These results could be explained as due to our measurements being performed in SW620 cell clusters, which were shown to be stiffer [30].

CLSM was used to visualize the differences in terms of cellular structures and function of the three cell types. Nuclei, actin filaments, tubulin, vinculin, total Fak, and focal adhesions were labeled (Figure 6, Figure 7, Figure 8 and Figure 9). Total FAK includes the free FAK distributed inside the cell and the p-FAK (also called the activated FAK) that integrates the focal adhesion complex [31]. In our work, analyzing Figure 9, it is possible to observe the presence of FAK superimposed with some parts of the cytoskeleton in the HCT15 cells, which may be part of the binding complex of the cells to the substrate, forming the focal adhesion. Tubulin is a protein that polymerizes into long chains that form microtubules. These structures can present different formations, which avoid the cells’ division in terms of mitosis, promoting cell death. The normal colon expresses little or no tubulin βII [32]. Ruksha et al. observed βII-tubulin expression in patients with CRC cancer. Those who presented more βII-tubulin amount had a poor prognosis [33]. Kaverina and Straube mentioned that microtubule-dependent alteration of cell migration implied changes in microtubule dynamics [34]. HCT15 presented considerably more cortical tubulin than HCT116 and SW620, which could be related to the migratory ability of such cells. However, different tubulin isotypes were not stained, so it is difficult to draw more conclusions in terms of mechanical and migratory properties.

Li et al. mentioned that vinculin expression was significantly downregulated in highly metastatic CRC cell lines, expressing EMT indicators. This loss of vinculin can be used as a prognostic factor for CRC [35]. Focal adhesions (FAs) are protein complexes that bind the cytoskeleton to the ECM or substrate via the integrin receptors present at the cellular membrane. Several proteins constitute the FAs, such as FAK and talin, both found at the end of stress fibers. The function of FAs is to transmit the force internally generated by the cytoskeleton to the ECM or substrate, and vice versa, via adhesion receptors [36]. FAK is one of the key signaling molecules regulating cell adhesion, migration, and survival [37]. It is localized in the focal adhesion complexes formed at the cytoplasmic side of the cell attachment to the ECM or substrates, being activated after force generation via actomyosin fibers attached to this complex [37].

SW620 cells presented more F-actin, which could be explained by their layer-by-layer growth, i.e., F-actin filaments in multiple cells were piled and hence superimposed. This observation is most likely responsible for the highest elastic modulus values in the SW620 cell line when compared to the HCT15 and HCT116 cell lines. Xu et al. compared different cancer cells, including HCT116, in terms of expression of p-FAK, through the inhibition of sphingosine kinase 1 (SphK1) since this enzyme plays an important role in CRC metastasis [38]. They observed that the inhibition of SphK1 reduced p-FAK expression, thus decreasing cell mobility. In Figure 8 and Figure 9, FAK can be observed and co-localized with F-actin, mainly in HCT116, which could indicate the presence of FAs and, together with the filopodia formation, as well as the presence of vinculin, explain the migratory behavior of HCT116. The filopodia formation and vinculin were not present in the SW620 cell line, which can support the hypothesis of lower migration of the SW620 cell lines. In the case of vinculin, Humphries et al. mentioned that it could regulate integrin dynamics, regulating the mechanotransduction force machinery, contributing to binding sites inducing a great focal adhesion growth through the direct interaction with actin and talin [39]. Albuquerque-González et al. studied the effect of migrastatin and imipramine inhibition of lamellipodia protrusion in HCT116 cells [40]. In the case of present HCT116 cells, their migratory behavior can thus be possibly attributed to lamellipodia formation.

Regarding cell migration (see Supplementary Material, Figure S2), only the HCT116 cells closed the gap completely, while it took 96 h for the HCT15 cells to completely close the gap. The SW620 metastatic cells did not close the gap, not even after 144 h (6 days). In fact, SW620 cells could only close the gap by 50% at 120 h. The results obtained for SW620 cells are in accordance with AFM and CSLM assays. The SW620 metastatic cell line is the stiffest compared with the HCT15 and HCT116 cell lines. In the CLSM images, the SW620 cell line did not present vinculin. The more metastatic these cells are, the less vinculin expression they present. Vinculin contributes to the stabilization of focal adhesion under high forces through the regulation of contractile stress generation, influencing cell migration speed [41,42]. The cells deficient in vinculin do not form lamellipodia and accumulate stress fibers or extent over the substrate; only filopodia is formed [43]. As in ovarian cancer, the loss of vinculin expression in ovarian cancer cells predicts a poor prognosis for this cancer type [44]. Targeting FAK is considered promising to avoid cancer progression, inhibiting mechanisms such as cancer migration and invasion and enhancing therapeutic strategies for CRC [45].

Some works have been published using the AFM as a powerful tool to distinguish healthy states from different pathological states in cells and tissues [8,46]. This technique could have a practical application in the surgery room to help the surgical team to distinguish tumor tissues from healthy ones through the measurement in loco of the stiffness. This procedure could avoid more surgeries on the same patient/the same tumor, avoiding physical and psychological pain and extra costs to the health system.

## 5. Conclusions

This work had the objective of comparing three CRC cell lines, namely, HCT116, HCT15, and SW610, in terms of their mechanical properties, structural features, and cell migration. All cells were significantly different from each other in terms of apparent Young’s, storage, and loss moduli. In terms of power-law exponent (correlated with fluidity) and loss tangent, the HCT116 and SW620 cells were shown to not be significantly different. SW620 metastatic cell lines presented the highest elasticity (storage modulus), cortical and distributed F-actin filaments in several layers, fewer focal contacts, the absence of vinculin, and lessened migratory behavior. HCT15 cell lines presented a lower elasticity (storage modulus), high cortical tubulin, less cortical F-actin, less vinculin, and more focal adhesion contact points presenting filopodia, which could explain the migratory behavior. HCT116 cell lines presented elasticity (storage modulus) in between the SW620 and the HCT115 cell lines, with high cortical F-actin, a high amount of vinculin, less tubulin, and intermediate levels of total FAK, abundant filopodia formation, which is likely associated with the greatest migratory behavior. This study also contributed to the hypothesis that there may be mechanisms other than mechanical properties that must be evaluated, mainly in the case of the HCT15 cell line. In the present work, distinct types of tubulin were not stained and thus do not allow distinction between them, which could provide more detailed information to understand how and which tubulin filaments contribute to mechanical and migratory properties.

## Figures and Tables

**Table 1:** Values of median and percentiles of Young’s modulus, storage modulus, loss modulus, power-law exponent applied to the storage modulus, and loss tangent quantification determined by AFM of HCT15, HT116, and SW620 cell lines.

Mechanical Property	Cell Line								
	HCT15			HCT116			SW620		
	25th	Median	75th	25th	Median	75th	25th	Median	75th
Apparent Young’s modulus (kPa)	1.70	1.87	2.62	1.94	2.61	3.67	2.86	4.29	5.78
Storage modulus (kPa)	1.39	1.91	2.38	2.76	3.64	4.8	3.8	4.66	6.78
Loss modulus (kPa)	0.25	0.34	0.41	0.42	0.51	0.63	0.59	0.71	0.84
Power-law exponent of the storage modulus	0.11	0.12	0.15	0.08	0.09	0.11	0.07	0.08	0.10
Loss tangent	0.15	0.18	0.21	0.13	0.15	0.16	0.12	0.14	0.17

**Table 2:** Statistical data treatment of HCT15, HCT116, and SW620. The Wilcoxon Rank’s test was used. ^(^***^)^ Statistically significant differences between the distinct CRC cell lines (*p* < 0.001).

Cell Line	Apparent Young’s Modulus (kPa)	Storage Modulus (kPa)	Loss Modulus (kPa)	Power-Law Exponent of the Storage Modulus	Loss Tangent
HCT15/HCT116 ***	0	0	0	0	7.21 × 10^−12^
HCT15/SW620 ***	0	0	0	0	3.15 × 10^−7^
HCT116/SW620	0 ***	0.004 ***	1.7 × 10^−5^ ***	0.004	0.82

**Table 3:** Summary of results obtained from AFM, CLSM, and cell-migration assays of the three CRC cell lines.

Analyzed Parameters	Cell Lines		
	HCT15	HCT116	SW620
Storage modulus (kPa)	1.91	3.64	4.66
Loss modulus (kPa)	0.34	0.51	0.71
Power-law exponent of the storage modulus	0.12	0.09	0.08
Loss tangent	0.18	0.15	0.14
Cortical actin	Low amount (+)	Very well distributed (+++)	Defined (++)
Cortical tubulin	(+++)	(+)	(+)
Vinculin	(+)	Distributed in whole cytoplasm (+++)	(−)
Total FAK	At focal contact points (+)	Abundant and distributed through whole cytoplasm (++)	Abundant and distributed through whole cytoplasm and at focal contact points (+++)
Filopodia formation	(+++)	(+++)	(−)
Cell migration	Gap closed at 96 h (++)	Gap closed at 72 h (+++)	Did not close the gap at 144 h (−)

Legend: (−) absence; (+) low; (++) intermediate; (+++) high.

## Data Availability

The data can be shared up on request.

## Supplementary Materials

The following supporting information can be downloaded at: https://www.mdpi.com/article/10.3390/cancers14205053/s1, Figure S1: Example of a power spectral density (PSD) with the thermal fits using single harmonic oscillator (SHO) and Lorentz’s algorithms, for the cantilever model PFQNM-LC-A-CAL. For both algorithms, the corrections factor was calculated. The thermal data are shown in red, in which the maximum amplitude peak corresponds to 20 kHz. The fit of the thermal with the SHO algorithm is represented by the green line. The Lorentzian fit of the PSD is presented by the blue line. It was observed that the Lorentz’s algorithm fitted better the thermal data when compared with the SHO fitting, as can be seen when analyzing at the noise level away from the peak, e.g., below 3 or above 50 kHz. Figure S2: Cell migration assay of HCT15, HCT116 and SW620 cell lines. (a) Time-lapse images of the gaps after 0, 24, 48, 72, 96, 120, and 144 h. Scale bars correspond to 120 μm. (b) Cell migration assay quantification. Graph showing the cell-free gap closure over time. Dashed lines represent the standard error of the mean of three independent.
