# Supplementary material for: Mechanical Properties of Colorectal Cancer Cells Determined by Dynamic Atomic Force Microscopy: A Novel Biomarker
Source: Cancers (Basel). 2022 Oct 15;14(20):5053. doi: 10.3390/cancers14205053 (PMC9600571; doi:10.3390/cancers14205053)
Supplement: Supplementary file 1 [file cancers-14-05053-s001.zip › cancers-1910212-supplementary.pdf]

Supplementary materials

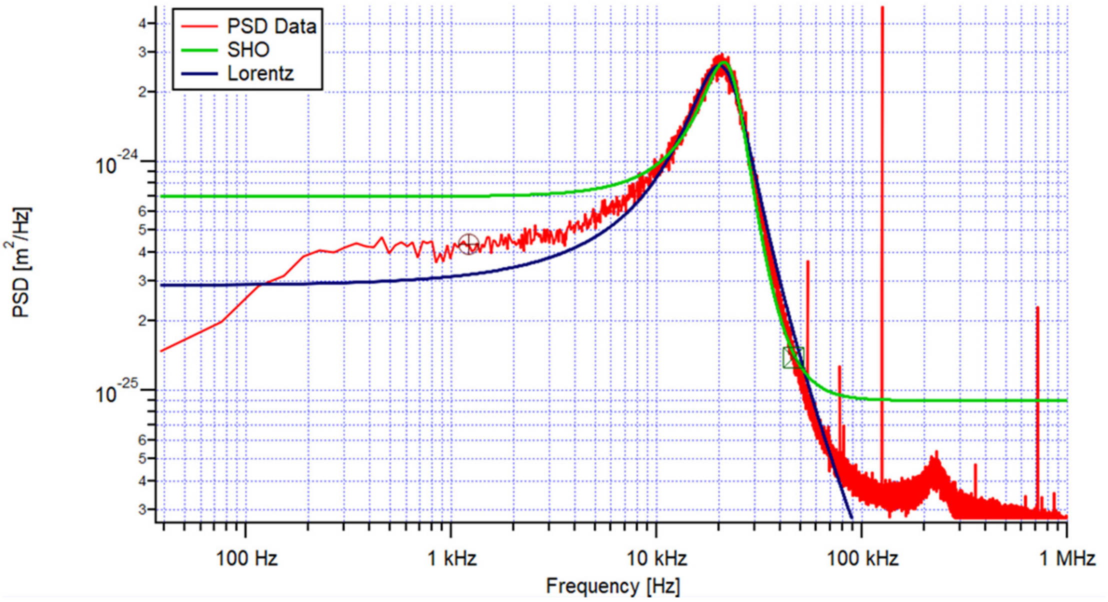

Figure S1. Example of a power spectral density (PSD) with the thermal fits using single harmonic oscillator (SHO) and Lorentz's algorithms, for the cantilever model PFQNM-LC-A-CAL. For both algorithms, the corrections factor was calculated. The thermal data are shown in red, in which the maximum amplitude peak corresponds to 20 kHz. The fit of the thermal with the SHO algorithm is represented by the green line. The Lorentzian fit of the PSD is presented by the blue line. It was observed that the Lorentz's algorithm fitted better the thermal data when compared with the SHO fitting, as can be seen when analyzing at the noise level away from the peak, e.g. below 3 or above 50 kHz.

(a)

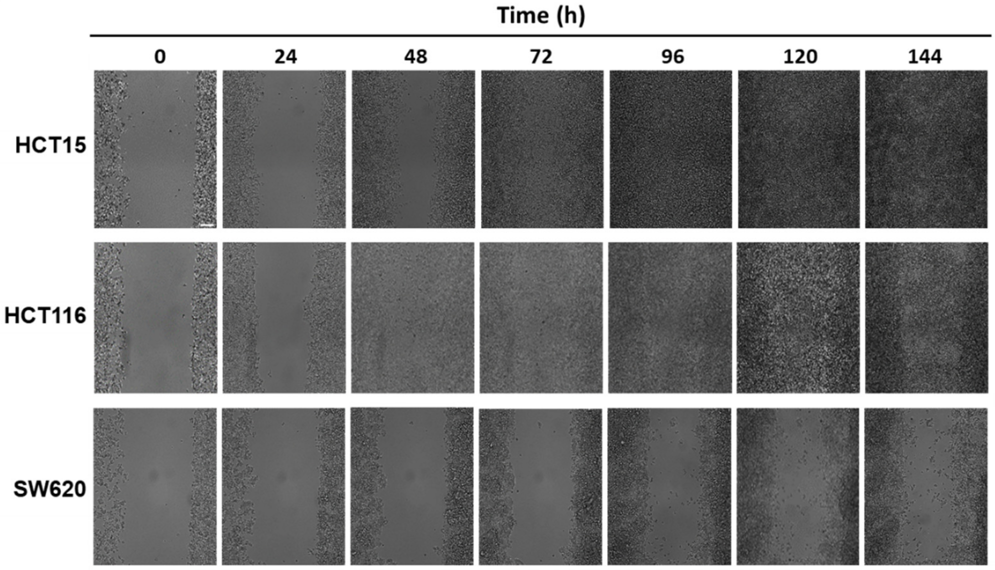

(b)

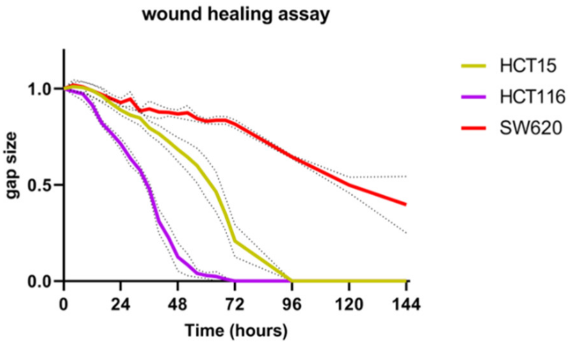

Figure S2. Cell migration assay of HCT15, HCT116 and SW620 cell lines. (a) Time-lapse images of the gaps after 0, 24, 48, 72, 96, 120, and 144 h. Scale bars correspond to 120 µm. (b) Cell migration assay quantification. Graph showing the cell-free gap closure over time. Dashed lines represent the standard error of the mean of three independent.
